# ATG7 Overexpression Is Crucial for Tumorigenic Growth of Bladder Cancer In Vitro and In Vivo by Targeting the ETS2/miRNA196b/FOXO1/p27 Axis

**DOI:** 10.1016/j.omtn.2017.04.012

**Published:** 2017-04-14

**Authors:** Junlan Zhu, Yang Li, Zhongxian Tian, Xiaohui Hua, Jiayan Gu, Jingxia Li, Claire Liu, Honglei Jin, Yulei Wang, Guosong Jiang, Haishan Huang, Chuanshu Huang

**Affiliations:** 1 Zhejiang Provincial Key Laboratory for Technology and Application of Model Organisms, School of Life Sciences, Wenzhou Medical University, Wenzhou, Zhejiang 325035, China; 2 Nelson Institute of Environmental Medicine, New York University School of Medicine, Tuxedo, NY 10987, USA

**Keywords:** ATG7, bladder cancer, tumorigenic growth, miR-196b, FOXO1, p27

## Abstract

Human bladder cancer (BC) is the fourth most common cancer in the United States. Investigation of the strategies aiming to elucidate the tumor growth and metastatic pathways in BC is critical for the management of this disease. Here we found that ATG7 expression was remarkably elevated in human bladder urothelial carcinoma and N-butyl-N-(4-hydroxybutyl)nitrosamine (BBN)-induced mouse invasive BC. Knockdown of ATG7 resulted in a significant inhibitory effect on tumorigenic growth of human BC cells both in vitro and in vivo by promoting p27 expression and inducing cell cycle arrest at G2/M phase. We further demonstrated that knockdown of ATG7 upregulated FOXO1 (forkhead box protein O 1) expression, which specifically promoted *p27* transcription. Moreover, mechanistic studies revealed that inhibition of ATG7 stabilized *ETS2* mRNA and, in turn, reduced *miR-196b* transcription and expression of *miR-196b*, which was able to bind to the 3′ UTR of *FOXO1* mRNA, consequently stabilizing *FOXO1* mRNA and finally promoting *p27* transcription and attenuating BC tumorigenic growth. The identification of the ATG7/FOXO1/p27 mechanism for promoting BC cell growth provides significant insights into understanding the nature of BC tumorigenesis. Together with our most recent discovery of the crucial role of ATG7 in promoting BC invasion, it raises the potential for developing an ATG7-based specific therapeutic strategy for treatment of human BC patients.

## Introduction

Bladder cancer (BC) is a significant public health issue worldwide; it is the fourth most commonly diagnosed tumor and the second most common cause of death among genitourinary tumors.^1^ Unfortunately, current conventional clinical and pathological parameters are unable to accurately predict outcomes or determine effective treatment strategies. The advances in suitable therapies for increasing the survival rate of BC patients have been limited because of the poor understanding of the underlying mechanisms of tumorigenesis and cancer progression. Therefore, the identification of novel therapeutic targets to improve the outcome of patients with BCs is highly critical.

ATG7 (autophagy-related gene 7) is an E1-like activating enzyme involved in the two ubiquitin-like systems required for autophagy.^2^, ^3^ It has been reported that the liver-specific deletion of ATG7 promotes spontaneous tumorigenesis by simultaneous deletion of p62.^4^ However, lung-specific ATG7 deficiency inhibits Kras^G12D^-driven lung tumor growth and reduction of lung tumor burden with dysfunctional mitochondria and proliferative arrest.^5^ ATG7 deficiency produces an autophagy-deficient phenotype and inhibits PTEN (phosphatase and tensin homolog)-deficient prostate tumor progression through management of protein homeostasis under endoplasmic reticulum (ER) stress.^6^ Additionally, in our most recent studies, we have shown that ATG7 and its mediated autophagy were much higher in human BC cell lines and N-Butyl-N-(4-hydmoxybutyl)nitrosamine (BBN)-treated UROtsa cells as well as in BBN-induced mouse invasive BCs, which play a promotive role in human BC invasion (J.Z., unpublished data). However, the exact natural contribution of ATG7 to BC growth has not been explored yet. Here we elucidated the potential role and molecular mechanism of the essential autophagy gene ATG7 in promoting human BC tumorigenic growth in vitro and in vivo.

p27 is a cyclin-dependent kinase (CDK) inhibitor that negatively regulates cell proliferation through the inhibition of cell cycle progression.^7^ In a quiescent state, the p27 protein mainly presents in the nucleus and binds to the cyclin/CDK complex to inhibit cell cycle progression.^8^ In cancer cells, p27 is inactivated through multiple mechanisms, including impaired synthesis, accelerated degradation, and mislocalization.^9^ Studies of gene deletion have shown that mice lacking p27 develop multiorgan hyperplasia and tumorigenesis.^10^ In a BC chemical carcinogenesis model, *p27*^−/−^ mice develop BC at a much earlier time point than their wild-type counterparts because of its critical role in controlling urothelial proliferation.^11^ Moreover, our most recent studies demonstrate that the upregulation of *p27* transcription by FOXO1 (forkhead box protein O 1) is crucial for its inhibition of BC cell growth (G.J., unpublished data). In the current study, we show that the FOXO1/p27 axis is the ATG7 downstream mediator for promotion of BC tumorigenic growth. We found that ATG7 overexpression led to instability of *ETS2* mRNA, subsequently increasing *miR-196b* transcription, further inhibiting *FOXO1* mRNA stability by directly targeting its mRNA 3′ UTR, which, in turn, resulted in reduction of *p27* transcription and promoted G2/M transition and the tumorigenic growth of human BC.

## Results

### ATG7 Overexpression Promoted Human BC Tumorigenic Growth Both In Vitro and In Vivo

**Figure 1:**
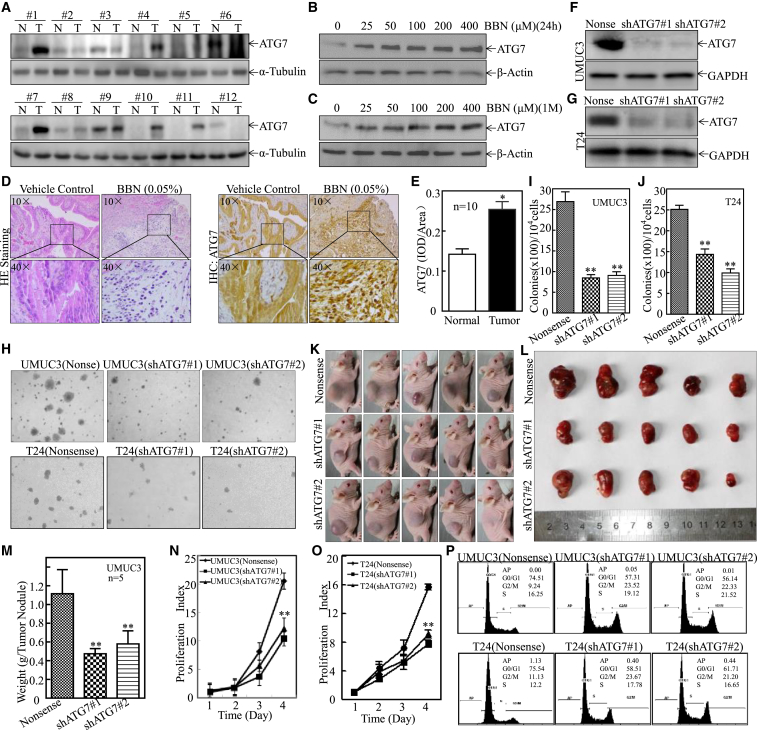
ATG7 Was Overexpressed in Human BCs, BBN-Induced Human Normal Bladder Urothelial UROtsa Cells, and BBN-Induced Highly Invasive Mouse BCs and Was Crucial for Anchorage-Independent Growth In Vitro and Tumorigenicity of Human BC Cells In Vivo (A) Total protein lysates were prepared from human bladder cancerous tissues (T) and paired adjacent normal tissues (N) among 12 patients diagnosed with invasive BC and subjected to western blot analysis for determining the ATG7 protein expression profile. (B and C) Human normal bladder urothelial cell line UROtsa cells were treated with BBN at different doses for 24 hr (B) or for 1 month (C). The total cell lysates were subjected to western blot to determine the expression of ATG7. β-Actin was used as a protein loading control. (D) H&E staining and IHC staining were performed to evaluate morphology and ATG7 expression in BBN-induced mouse invasive BCs. The IHC images were captured using the AxioVision Rel.4.6 computerized image system. (E) The ATG7 protein expression levels were analyzed by calculating the integrated IOD/area using Image-Pro Plus version 6.0. Three independent experiments were performed, and Student’s t test was utilized to determine the p values. An asterisk indicates a significant increase from vehicle-treated mice (*p < 0.05). (F and G) ATG7 knockdown constructs were stably transfected into UMUC3 (F) and T24 (G) cells, respectively. The knockdown efficiency of ATG7 protein was assessed by western blotting. (H) UMUC3(shATG7#1) cells, UMUC3(shATG7#2) cells versus UMUC3(Nonsense) cells or T24(shATG7#1) cells, and T24 (shATG7#2) cells versus T24 (Nonsense) cells were subjected to an anchorage-independent soft agar assay using the protocol described in Materials and Methods. Representative images of colonies of the indicated cells were taken under an Olympus DP71. (I and J) The number of colonies was counted with more than 32 cells of each colony, and the results are presented as colonies per 10^4^ cells. The bars show mean ± SD from three independent experiments. Double asterisks indicate a significant decrease in comparison with nonsense control transfectants (**p < 0.05). (K–M) Athymic nude mice were subcutaneously injected with UMUC3(Nonsense), UMUC3(shATG7#1), and UMUC3(shATG7#2) transfectants (2 × 10^6^ suspended in 100 μL PBS) into the right axillary region as described in Materials and Methods. Four weeks after cell injection, the mice were sacrificed, and the tumors were surgically removed and photographed. (K and L) as well as weighed (M). Bars represent mean ± SD from each group of five mice. Student’s t test was utilized to determine the p value. Double asterisks indicate a significant decrease in comparison with UMUC3(Nonsense) transfectants (**p < 0.05). (N and O) UMUC3 transfectants and T24 transfectants, as indicated, were plated in 96-well plates at a density of 5,000 cells/well. The cell culture medium was then replaced with 0.1% FBS DMEM or 0.1% FBS DMEM-F12 (1:1) and cultured for 12 hr. The medium was replaced with normal medium and cultured for another 1, 2, 3, or 4 days. Subsequently, an ATP activity assay was performed using the protocol described in Materials and Methods. Double asterisks indicate a significant decrease from the nonsense control. (P) The indicated cells (2 × 10^5^) were seeded into a 6-well plate and cultured overnight. Following synchronization in 0.1% FBS for 12 hr, the medium was replaced with 10% FBS DMEM or 5% FBS DMEM-F12 (1:1) for another 12 hr, and then the cells were stained with propidium iodide for cell cycle analysis as described in Materials and Methods. The results represent one of three independent experiments.

Our most recent studies have shown that ATG7 and its mediated autophagy play a positive role in the promotion of BC cell invasion by elevation of RhoGDIβ protein expression. To evaluate whether ATG7 also regulates BC growth, we first detected ATG7 expression in human BC tissues and found that it was overexpressed in 66.7% (8 of 12) of human BCs in comparison with their adjacent normal bladder tissues (Figure 1A). BBN is a genotoxic carcinogen that is widely used in animal bladder carcinogenesis studies.^12^, ^13^, ^14^ The bladder tumors induced by BBN exposure in mice are able to mimic human BCs.^15^, ^16^, ^17^ Our most recent studies indicate that human normal bladder urothelial UROtsa cells repeatedly exposed to BBN at 400 μM for over 6 months gain the capability of anchorage-independent growth in soft agar, a hallmark of cellular malignant transformation, without showing any observable cytotoxicity (H.J., unpublished data). Thus, the potential effect of BBN on ATG7 expression in an in vitro cell culture model and an in vivo mouse bladder carcinogenic model were further evaluated. As shown in Figures 1B and 1C, ATG7 upregulation was observed in 24-hr or 1-month BBN-treated UROtsa cells in a dose- and time-dependent fashion. Consistent with this observation in the in vitro-cultured cell model, ATG7 overexpression was also observed in BBN-induced mouse BCs in vivo, as demonstrated by immunohistochemistry (IHC) staining (n = 10) (Figures 1D and 1E). Our results consistently demonstrate that elevation of ATG7 expression is observed in human BCs and BBN-treated UROtsa cells in vitro as well as in BBN-induced highly invasive BC tissues in vivo.

To understand the biological role of ATG7 in regulating BC cell proliferation and tumorigenic growth, small hairpin RNA specifically targeting human ATG7 (shATG7) was stably transfected into UMUC3 and T24 human BC cells, respectively (Figures 1F and 1G). Knockdown of ATG7 inhibited BC cell anchorage-independent growth in both UMUC3 and T24 cells (Figures 1H–1J). To further determine the potent oncogenic activity of ATG7 in vivo, UMUC3(shATG7#1), UMUC3(shATG7#2), and UMUC3(Nonsense) cells were subcutaneously injected into nude mice. Unexpectedly, the knockdown of ATG7 dramatically inhibited UMUC3 xenograft tumor growth and reduced the tumor burden (weight) compared with the UMUC3(Nonsense) group (p < 0.01, n = 5) (Figures 1K–1M). Consistent with the tumorigenic growth in vivo, knockdown of ATG7 dramatically inhibited the monolayer growth of UMUC3 and T24 cells, accompanied by the induction of G2/M phase arrest in UMUC3 and T24 cells in vitro (Figures 1N–1P). Taken together, these results demonstrate a novel positive regulatory effect of ATG7 on human BC tumorigenic growth.

### ATG7 Promotion of Human BC Cells Tumorigenic Growth Was Mediated by Its Targeting of p27

**Figure 2:**
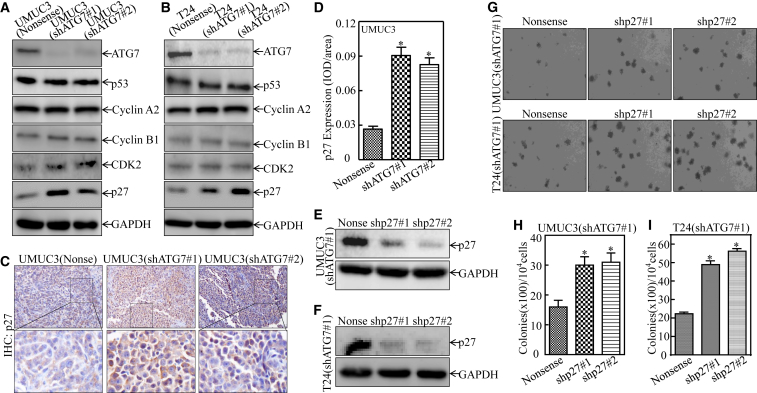
p27 Downregulation Mediates ATG7 Promotion of Human BC Anchorage-Independent Growth (A and B) The indicated cell extracts were subjected to western blot for determining the expression of ATG7, p53, Cyclin A2, Cyclin B1, CDK2, and p27. GAPDH (glyceraldehyde 3-phosphate dehydrogenase) was used as a protein loading control. (C and D) IHC staining was performed to evaluate p27 expression in xenograft tumors obtained from nude mice. The IHC images were captured using the AxioVision Rel.4.6 computerized image system, and protein expression levels were analyzed by calculating the integrated IOD/area using Image-Pro Plus version 6.0. Results are presented as the mean ± SD of five mice in each group. Student’s t test was utilized to determine the p value (*p < 0.05). (E and F) p27 knockdown constructs were stably transfected into UMUC3(shATG7#1) and T24(shATG7#1) cells. The knockdown efficiency of p27 protein was assessed by western blotting. (G) UMUC3(shATG7#1/shp27#1) cells, UMUC3(shATG7#1/shp27#2) cells versus UMUC3(shATG7#1/Nonsense) cells or T24(shATG7#1/shp27#1) cells, and T24(shATG7#1/shp27#2) cells versus T24(shATG7#1/Nonsense) cells, were subjected to an anchorage-independent soft agar growth assay using the protocol described in Materials and Methods. Representative images of colonies were captured under an Olympus DP71. (H and I) The number of colonies was counted, with the standard being more than 32 cells of each colony, and the results are presented as colonies/10^4^ cells. The bars show mean ± SD from three independent experiments. An asterisk indicates a significant increase in comparison with nonsense transfectants (*p < 0.05).

To elucidate the mechanism of ATG7 involved in the regulation of G2/M transition, western blotting was performed to screen the potential ATG7 downstream G2/M transition regulators. As shown in Figures 2A and 2B, knockdown of ATG7 specifically increased p27 protein abundance with no remarkable effect on the expression of p53, Cyclin A2, Cyclin B1, and CDK2 in both UMUC3 and T24 cells, suggesting that ATG7 overexpression provides an inhibitory effect on p27 expression in BC cells. Moreover, the inhibitory effect was also observed in in vivo xenograft tumors obtained from nude mice injected with UMUC3(shATG7#1) and UMUC3(shATG7#2) cells in comparison with mice injected with UMUC3(Nonsense) cells (Figures 2C and 2D). Thus, we anticipated that p27 might be an ATG7 downstream mediator responsible for induction of G2/M phase arrest and inhibition of human BC tumorigenic growth in BC cells. To test this notion, we stably transfected shRNA specifically targeting p27 (shp27#1 and shp27#2) into UMUC3(shATG7#1) cells (Figure 2E) and T24(shATG7#1) cells (Figure 2F). Their effect on anchorage-independent growth ability was evaluated, and the results showed that knockdown of p27 increased the anchorage-independent growth ability of UMUC3(shATG7#1) cells and T24(shATG7#1) cells compared with that observed in scramble nonsense transfectants (Figures 2G–2I), suggesting that p27 is an ATG7 downstream effector responsible for the promotion of human BC tumorigenic growth.

### ATG7 Inhibited p27 Transcription by Attenuating FOXO1 Protein Expression

**Figure 3:**
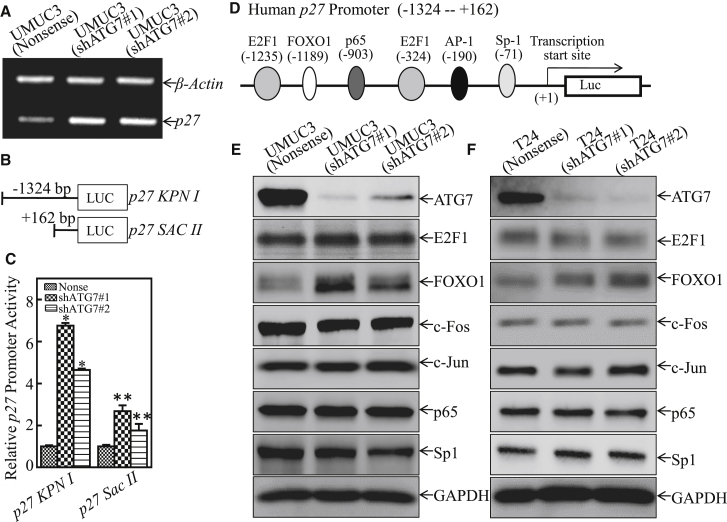
ATG7 Overexpression Inhibited *p27* mRNA Transcription in Human BC Cells (A) UMUC3(shATG7#1), UMUC3(shATG7#2), and UMUC3(Nonsense) cells were cultured in 6-well plates until the cell density reached 70%–80%. Following synchronization for 12 hr, the medium was replaced with 10% FBS DMEM for another 12 hr. Then the cells were extracted for total RNA with TRIzol reagent. RT-PCR was used to determine *p27* mRNA expression, whereas *β-actin* was used as an internal control. (B) Schematic representation of the *p27* promoter region *p27 KPNI* and its deletion fragment *SACII*. (C) *p27* promoter transcription activity was evaluated by using the two *p27* promoter-driven luciferase reporters shown in (B). The results were normalized by internal TK activity, and the bars show mean ± SD from three independent experiments. The asterisk indicates a significant increase in comparison with vector control transfectant (*p < 0.05), whereas double asterisks indicate a significant decrease in *p27 SACII* transfectant in comparison with *p27 KPNI* transfectant (**p < 0.05). (D) Potential transcriptional factor binding sites in the *p27* promoter region (–1324 to +162) were analyzed by using the TRANSFAC 8.3 engine online. (E and F) The indicated cell extracts were subjected to western blot for determination of the expression of ATG7, E2F1, FOXO1, c-Fos, c-Jun, p65, and Sp1. GAPDH was used as a protein loading control. The result represented one of three independent experiments.

**Figure 4:**
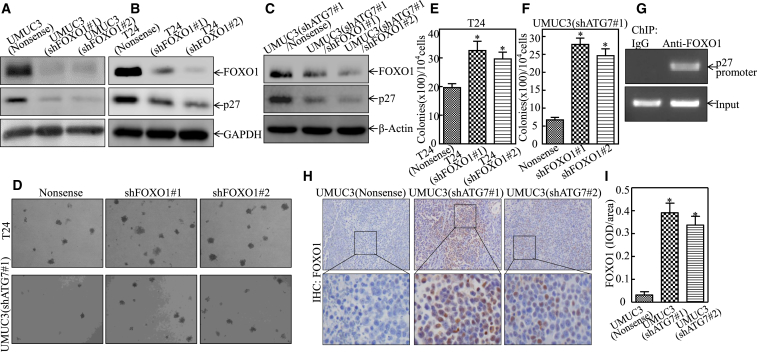
FOXO1 Is an ATG7-Regulated Transcription Factor Mediating p27 Downregulation in Human BCs In Vitro and In Vivo (A–C) FOXO1 knockdown constructs were stably transfected into UMUC3 (A), T24 (B), and UMUC3(shATG7#1) cells (C). The FOXO1 knockdown efficiency and its downstream p27 expression were assessed by western blotting. GAPDH or β-actin was used as a protein loading control. (D) The stable transfectants as indicated were subjected to an anchorage-independent soft agar growth assay. Representative images of colonies were photographed under an Olympus DP71. (E and F) The number of colonies was counted with more than 32 cells of each colony, and the results are presented as colonies per 10^4^ cells. The bars show mean ± SD from three independent experiments. The asterisk indicates a significant increase in comparison with nonsense transfectants as indicated (*p < 0.05). (G) A ChIP assay was carried out using anti-FOXO1 antibody to detect the interaction of FOXO1 with the p27 promoter. (H and I) IHC staining was performed to evaluate FOXO1 expression in the indicated xenograft tumors. The IHC images were captured using the AxioVision Rel.4.6 computerized image system, and protein expression levels were analyzed by calculating the integrated IOD/area using Image-Pro Plus version 6.0. Results are presented as the mean ± SD of five mice in each group. Student’s t test was utilized to determine the p value (*p < 0.05).

To investigate the mechanisms underlying the ATG7-downregulating p27 protein, we first examined *p27* mRNA levels. The results showed that *p27* mRNA was markedly upregulated in ATG7 knockdown transfectants (Figure 3A). Two different-length fragments of human *p27* promoter luciferase reporters, *p27 KPNI* and *p27 SACII*, as indicated in Figure 3B, were then transfected into UMUC3(Nonsense), UMUC3(shATG7#1), and UMUC3(shATG7#2) cells. The results showed that knockdown of ATG7 increased *p27 KPNI* promoter-driven reporter transcription activity in UMUC3 cells. However, knockdown of ATG7 had little effect on *p27 SACII* promoter activity (Figure 3C), revealing that the −1324 to +162 region of the promoter contains the binding site of the transcription factor(s) responsible for ATG7 inhibiting *p27* promoter transcription. Therefore, we bioinformatically analyzed the potential transcriptional factors that could bind to the −1324 to +162 region of the *p27* promoter (Figure 3D). Then we evaluated the expression of these transcription factors in both ATG7 knockdown cells and their scramble nonsense transfectants. The results showed that only FOXO1 exhibited high expression, whereas others showed no significant differences by knockdown of ATG7, suggesting that FOXO1 might be the effector for regulating p27 expression. To determine whether FOXO1 was the downstream mediator responsible for ATG7 promotion of human BC tumorigenic growth, shRNAs specifically targeting FOXO1 (shFOXO1#1 and shFOXO1#2) were stably transfected into UMUC3 cells, T24 cells, and UMUC3(shATG7#1) cells, as shown in Figures 4A–4C. Consistently, knockdown of FOXO1 attenuated p27 expression in all three types of transfectants, strongly revealing that FOXO1 is the critical transcription factor responsible for ATG7 inhibition of the transcription of the *p27* promoter. Moreover, the anchorage-independent growth ability was remarkably increased in FOXO1 knockdown T24 cells and UMUC3(shATG7) transfectants (Figures 4D–4F). The chromatin immunoprecipitation (ChIP) assay was carried out by using anti-FOXO1 antibody, and the results showed that FOXO1 could directly bind to the p27 promoter, as shown in Figure 4G. Significantly, ATG7 inhibition of FOXO1 protein expression was also observed in xenograft tumor tissues obtained from nude mice injected with UMUC3(shATG7) cells (Figures 4H and 4I). Collectively, our results strongly demonstrate that FOXO1 is the critical factor mediating ATG7 promotion of BC growth by directly binding to the p27 promoter, thereby inhibiting p27 expression.

### ATG7 Promoted *FOXO1* mRNA Degradation by Regulating *FOXO1* 3′ UTR of mRNA activity

**Figure 5:**
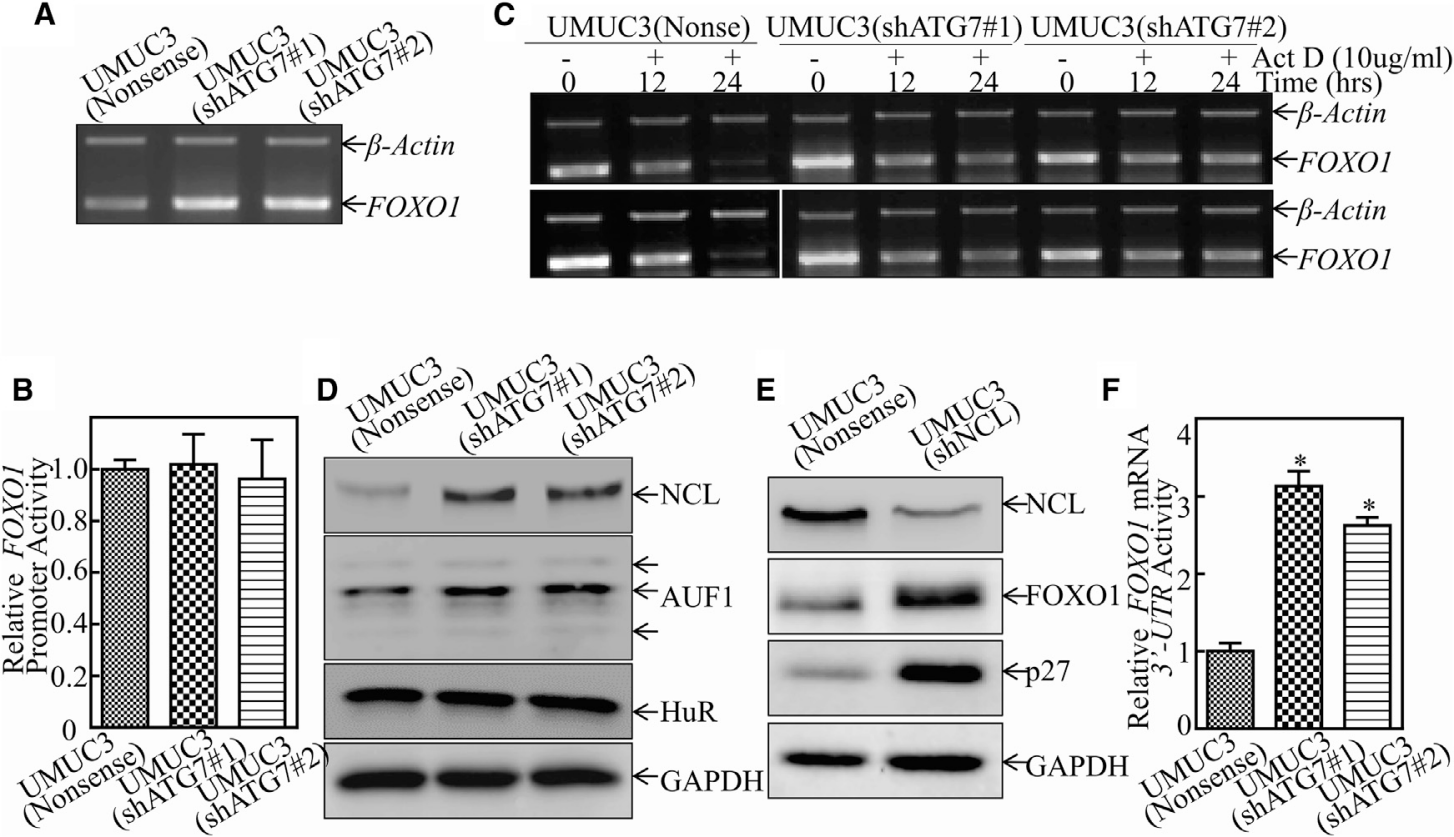
ATG7 Overexpression Decreased *FOXO1* mRNA Stabilization by Regulating Its mRNA 3′ UTR Activity (A) UMUC3(shATG7#1), UMUC3(shATG7#2), and UMUC3(Nonsense) cells were cultured in 6-well plates until the cell density reached 70%–80%. Following synchronization for 12 hr, the medium was then replaced with 10% FBS DMEM for another 12 hr. Then the cells were extracted for total RNA with TRIzol reagent. RT-PCR was used to determine *FOXO1* mRNA expression, and *β-actin* was used as an internal control. (B) The human *FOXO1* promoter-driven luciferase reporter was used to evaluate its promoter transcription activity in the indicated transfectants. The results were normalized by internal TK activity. (C) UMUC3(shATG7#1), UMUC3(shATG7#2), and UMUC3(Nonsense) cells were seeded into 6-well plates. After synchronization, the indicated cells were treated with Act D for the indicated times. Total RNA was then isolated and subjected to RT-PCR analysis for mRNA levels of *FOXO1*, and *β-actin* was used as an internal control. (D) The indicated cell extracts were subjected to western blot for determination of NCL, AUF1, and HuR protein expression. GAPDH was used as a protein loading control. (E) NCL knockdown constructs were stably transfected into UMUC3 cells. The knockdown efficiency of NCL protein and the expression of FOXO1 and p27 were evaluated by western blotting. GAPDH was used as a protein loading control. (F) The pMIR-*FOXO1* 3′ UTR mRNA reporter was transiently transfected into the indicated cells, and the luciferase activity of each transfectant was evaluated. The luciferase activity is presented as relative to nonsense transfectant, normalized by using pRL-TK as an internal control. The bars show mean ± SD from three independent experiments. The asterisk indicates a significant increase in UMUC3(shATG7) in comparison with nonsense transfectant (*p < 0.05).

To investigate the mechanisms underlying ATG7 inhibition of FOXO1 protein expression, we first tested the potential regulatory effect of ATG7 on the *FOXO1* mRNA level. The results showed that the *FOXO1* mRNA level was remarkably increased in UMUC3(shATG7) and T24(shATG7) cells (Figure 5A). Based on the results above, we next determined whether *FOXO1* mRNA was upregulated at the level of either transcription or mRNA stability. The results showed that *FOXO1* promoter-driven luciferase reporter activity was comparable among UMUC3(shATG7#1) and UMUC3(shATG7#2) cells and their scramble nonsense transfectants (Figure 5B), excluding the possibility of *FOXO1* transcriptional regulation by ATG7. We therefore exploited the possibility of ATG7 destabilization of *FOXO1* mRNA. Upon inhibition of new mRNA transcription with actinomycin D (Act D), *FOXO1* mRNA degradation rates in UMUC3(Nonsense) cells were much faster than in UMUC3(shATG7) cells (Figure 5C). These results reveal that ATG7 overexpression results in unstable *FOXO1* mRNA. It has been reported that NCL has four RNA-binding domains and can stabilize its targeted mRNA,^18^ whereas AUF1 is able to bind to AU-rich element (ARE)-mRNAs and promote its targeted mRNA degradation.^19^ HuR could selectively bind to and stabilize ARE-mRNAs.^20^ Thus, we tested whether those RNA-binding proteins were involved in the ATG7 downregulation of *FOXO1* mRNA stability. As the data show in Figure 5D, HuR protein expression was comparable, whereas NCL and AUF1 expression was elevated in UMUC3(shATG7) cells compared with UMUC3(Nonsense) cells. Therefore, we excluded the participation of HuR and AUF1 in ATG7 destabilization of *FOXO1* mRNA. We next used NCL shRNA to establish stable transfectants in UMUC3 cells, creating UMUC3(shNCL) cells (Fig. 5E). Knockdown of NCL profoundly elevated the expression of both FOXO1 and p27, which is inconsistent with our anticipation that NCL inhibition by ATG7 mediates the reduction of FOXO1 and p27. The 3′ UTR of mRNA, has been reported to be involved in the regulation of its mRNA stability.^21^ Further results showed that knockdown of ATG7 significantly promoted *FOXO1* mRNA 3′ UTR activity (Figure 5F), revealing that the 3′ UTR of *FOXO1* mRNA might be involved in ATG7 inhibition of *FOXO1* mRNA stability in human BC cells.

### ATG7 Overexpression Upregulated miR-196b and Subsequently Destabilized *FOXO1* mRNA through Directly Binding to Its 3′ UTR of mRNA

**Figure 6:**
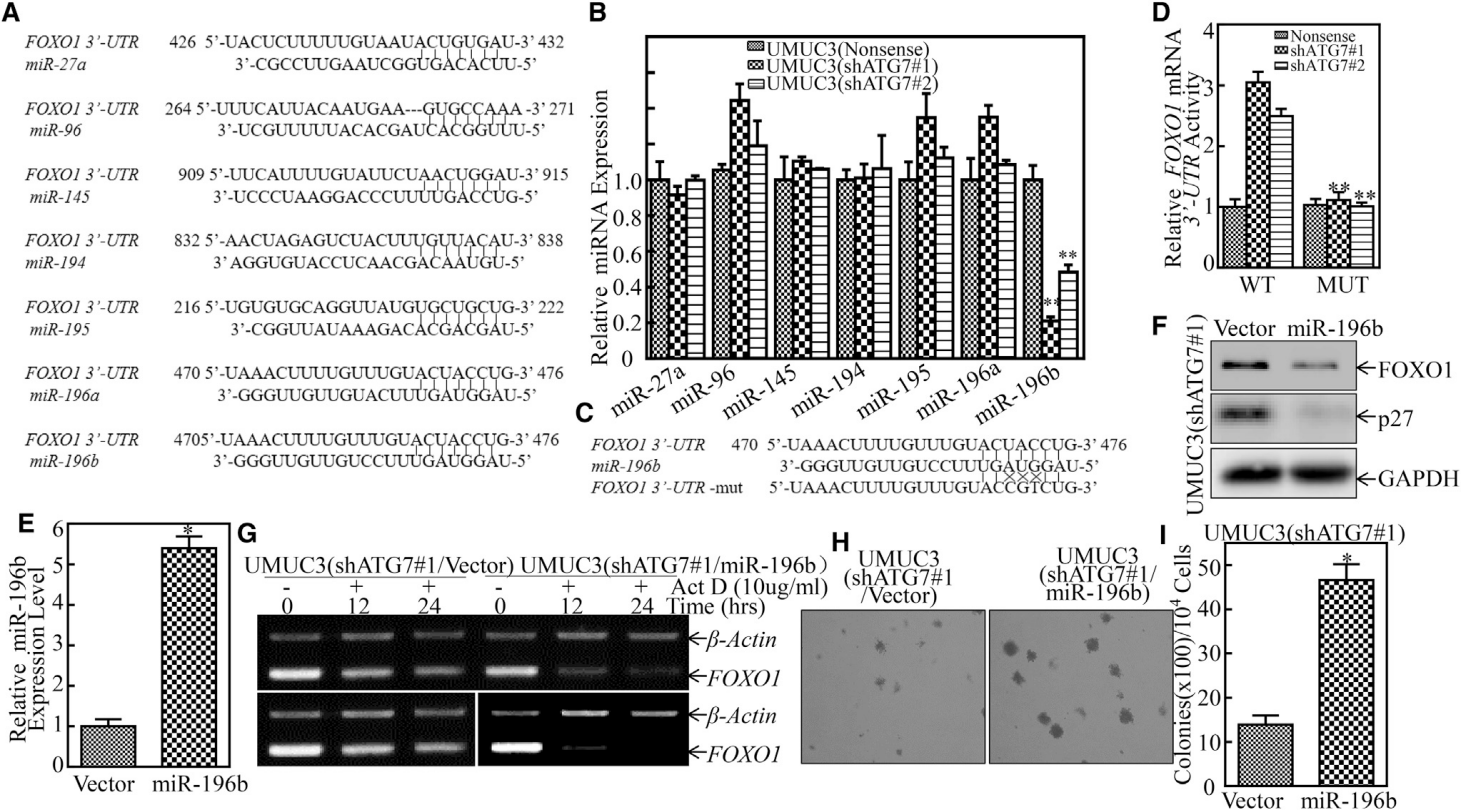
miR-196b Is an ATG7 Downstream Effector Responsible for Destabilization of *FOXO1* mRNA via Direct Binding to *FOXO1* mRNA 3′ UTR (A) The potential microRNA binding sites in *FOXO1* mRNA 3′ UTR were predicted by the TargetScan, Pictar, and miRANDA databases. (B) Quantitative real-time PCR was carried out to determine the expression of miRNAs in the indicated cells. The bars show mean ± SD from three independent experiments. Double asterisks indicate a significant decrease in comparison with UMUC3(Nonsense) cells (**p < 0.05). (C) Schematic of the construction of the *FOXO1* mRNA 3′ UTR luciferase reporter; its mutants were aligned with miR-196b. (D) Wild-type and mutant *FOXO1* 3′ UTR mRNA luciferase reporters were transiently co-transfected with pRL-TK into the indicated cells. The luciferase activity of each transfectant was evaluated, and the results are presented as relative *FOXO1* 3′ UTR mRNA activity. The bars show mean ± SD from three independent experiments. Double asterisks indicate a significant inhibition of 3′ UTR mRNA activity in the mutant transfectant in comparison with the mutant of the WT *FOXO1* 3′ UTR mRNA luciferase reporter transfectant (**p < 0.05). (E) miR-196b constitutively expressed plasmids were stably transfected into UMUC3(shATG7#1) cells. The stable transfectants were identified by real-time PCR. Bars represent mean ± SD from three independent experiments. Student’s t test was utilized to determine the p value. An asterisk indicates a significant increase in comparison with the scramble vector transfectant (*p < 0.05). (F) Cell lysates extracted from the indicated cells were subjected to western blot for determining the protein expression of FOXO1 and p27. GAPDH was used as a loading control. (G) UMUC3(shATG7#1/miR-196b) cells and their scramble vector transfectant were seeded into 6-well plates. After synchronization, the cells were treated with Act D for the indicated times. Then total RNA was isolated and subjected to RT-PCR analysis for mRNA levels of *FOXO1*, and *β-actin* was used as an internal control. (H) UMUC3(shATG7#1/miR-196b) cells and UMUC3(shATG7#1/Vector) cells were subjected to an anchorage-independent soft agar growth assay using the protocol described in Materials and Methods. Representative images of colonies were taken under an Olympus DP71. (I) The number of colonies was counted, with the standard being more than 32 cells of each colony, and the results are presented as colonies per 10^4^ cells. The bars show mean ± SD from three independent experiments. An asterisk indicates a significant increase in UMUC3(shATG7/miR-196b) cells in comparison with UMUC3(shATG7/Vector) cells (*p < 0.05).

MicroRNAs (miRNAs) are small (∼22-nt) non-coding RNAs that regulate gene expression by targeting its bound mRNA for post-transcriptional regulation.^22^ Based on the results shown above, we anticipated that miRNAs might be involved in the ATG7 regulation of *FOXO1* mRNA 3′ UTR activity. A bioinformatics search for putative miRNAs that could potentially target the 3′ UTR of *FOXO1* mRNA was performed by using TargetScan,^23^ Pictar,^24^ and miRANDA.^25^ We next carried out real-time PCR to evaluate the expression of the miRNAs, as indicated in Figure 6A, among UMUC3(Nonsense), UMUC3(shATG7#1), and UMUC3(shATG7#2) cells. As shown in Figure 6B, miR-196b was identified to be the only one that was downregulated in UMUC3(shATG7) cells. To test whether miR-196b is able to directly bind to the 3′ UTR of *FOXO1* mRNA for destabilization of *FOXO1* mRNA, the mutation of the miR-196b binding site in the *FOXO1* 3′ UTR of mRNA luciferase reporter was constructed as indicated in Figure 6C. Further results showed that *FOXO1* 3′ UTR of mRNA luciferase activity was significantly increased in UMUC3(shATG7) cells compared with that in UMUC3(Nonsense) cells, whereas the mutation luciferase reporter completely attenuated the responses of UMUC3 cells because of ATG7 knockdown (Figure 6D). These results demonstrate that the direct binding of miR-196b to the 3′ UTR of *FOXO1* mRNA is crucial for miR-196b destabilization of *FOXO1* mRNA. To define the effect of miR-196b on the regulation of FOXO1 expression, a construct expressing miR-196b was transfected into UMUC3(shATG7#1) cells, as shown in Figure 6E. In comparison with scramble vector transfectants, ectopic expression of miR-196b resulted in the inhibition of FOXO1 and p27 protein expression (Figure 6F). Further, the ectopic expression of miR-196b markedly promoted the degradation of *FOXO1* mRNA (Figure 6G) and promoted the anchorage-independent growth of UMUC3(shATG7#1/miR-196b) cells in comparison with UMUC3(shATG7#1/Vector) cells (Figures 6H and 6I). Taken together, our results demonstrate that ATG7 overexpression promotes miR-196b expression, which subsequently binds to the *FOXO1* mRNA 3′ UTR and leads to *FOXO1* mRNA degradation and *p27* transcription inhibition, in turn promoting human BC tumorigenic growth.

### ATG7 Promoted *miR-196b* mRNA Transcription by Destabilizing *ETS2* mRNA

**Figure 7:**
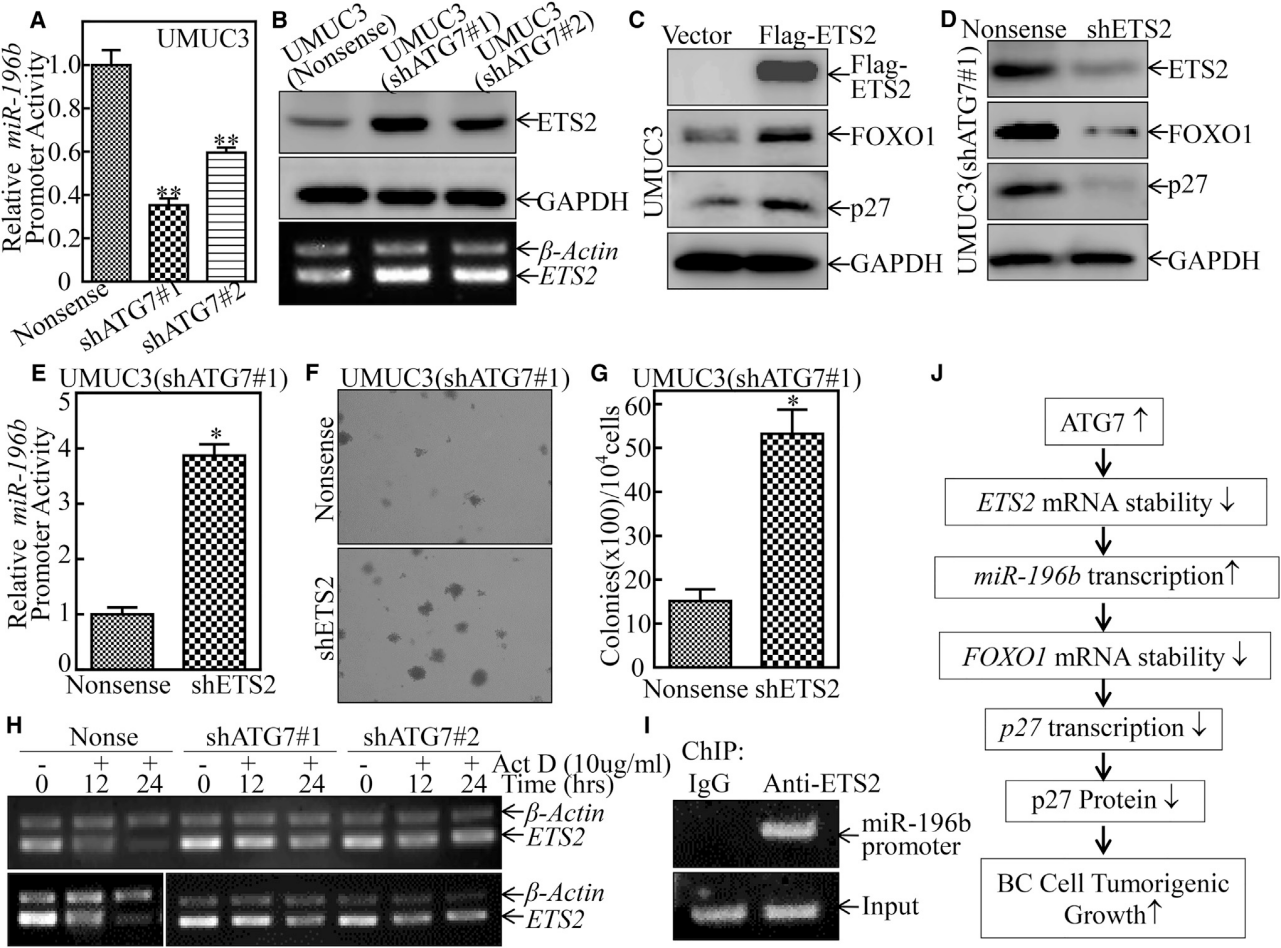
ATG7 Mediates *miR-196b* mRNA Transcription by Promoting *ETS2* mRNA Degradation in Human BC Cells (A) The *miR-196b* promoter-driven luciferase reporter, together with the TK reporter, was transiently transfected into UMUC3(Nonsense), UMUC3(shATG7#1), and UMUC3(shATG7#2) cells. The luciferase activity of each transfectant was evaluated, and the bars show mean ± SD from three independent experiments. Double asterisks indicate a significant decrease compared with nonsense control transfectants (**p < 0.05). (B) Cell lysates and total RNAs extracted from the cells as indicated were subjected to either western blot (top) for determination of protein expression of ETS2 or RT-PCR (bottom) for determination of *ETS2* mRNA expression, respectively. GAPDH and *β-actin* were used as loading controls. (C and D) The overexpressed FLAG-ETS2 plasmid and shRNA specifically targeting ETS2 were stably transfected into UMUC3 cells or UMUC3(shATG7#1) cells, respectively, and the cell extracts were then subjected to western blot for determination of FLAG, ETS2, FOXO1, and p27 expression. GAPDH was used as a protein loading control. (E) The *miR-196b* promoter-driven luciferase reporter, together with the TK reporter, was transfected into the indicated cells. The luciferase activity of each transfectant was evaluated, and the bars show mean ± SD from three independent experiments. An asterisk indicates a significant increase compared with nonsense transfectant (*p < 0.05). (F and G) UMUC3(shATG7#1/shETS2) cells versus UMUC3(shATG7#1/Nonsense) cells were subjected to an anchorage-independent soft agar growth assay using the protocol described in Materials and Methods. Representative images of colonies of the indicated cells were taken under an Olympus DP71. The number of colonies was counted, with the standard being more than 32 cells of each colony, and the results are presented as colonies per 10^4^ cells. The bars show mean ± SD from three independent experiments. An asterisk indicates a significant increase of colonies in UMUC3(shATG7#1/shETS2) cells in comparison with UMUC3(shATG7#1/Nonsense) cells (*p < 0.05). (H) UMUC3(shATG7#1) cells and UMUC3(shATG7#2) cells versus UMUC3(Nonsense) cells were seeded into 6-well plates. After synchronization, the indicated cells were treated with Act D for the indicated times. Then total RNA was isolated and subjected to RT-PCR analysis for mRNA levels of *ETS2*, and *β-actin* was used as an internal control. (I) 1 × 10^6^ UMUC3 cells were seeded into a 10-cm dish. After the cell density reached 80∼90%, a ChIP assay was performed with anti-ETS2 antibody to determine ETS2 binding to the motifs between −851 and −683 bp of the miR-196b promoter as described in Materials and Methods. (J) The proposed mechanisms underlying ATG7 overexpression in the promotion of human BC cell tumorigenic growth. ATG7 overexpression destabilizes *ETS2* mRNA, which further promotes the transcription of *miR-196b*, in turn reducing *FOXO1* mRNA stability and protein expression, and finally inhibiting *p27* mRNA transcription and protein expression and increasing tumorigenic growth of human BC cells.

To explore the mechanisms underlying ATG7 upregulation of miR-196b expression, we first evaluated the *miR-196b* promoter activity in UMUC3(Nonsense), UMUC3(shATG7#1), and UMUC3(shATG7#2) cells. As shown in Figure 7A, knockdown of ATG7 reduced *miR-196b* promoter activity. Recently, Liao et al. ^26^ demonstrated that knockdown of ETS2 expression increases miR-196b transcription and affects the invasion of gastric cancer cells. As expected, ETS2 was responsible for ATG7 inhibition of miR-196b transcriptional activity, and both ETS2 protein and mRNA levels were upregulated in ATG7 knockdown transfectants (Figure 7B). To further test the role of ETS2, FLAG-ETS2 and shRNA of ETS2 were stably transfected into UMUC3 cells and UMUC3(shATG7#1) cells, respectively (Figures 7C and 7D). The results showed that knockdown of ETS2 remarkably promoted *miR-196b* promoter-driven luciferase reporter transcriptional activity (Figure 7E), demonstrating that ETS2 does act as an inhibitory transcription factor of the *miR-196b* promoter. Consistently, knockdown of ETS2 dramatically impaired FOXO1 and p27 expression, whereas ETS2 overexpression increased FOXO1 and p27 protein expression (Figures 7C and 7D). Furthermore, the anchorage-independent growth of UMUC3(shATG7#1/shETS2) was elevated compared with UMUC3(shATG7#1/Nonsense) cells (Figures 7F and 7G). To elucidate the underlying mechanism of ATG7 inhibition of *ETS2* mRNA, we evaluated the mRNA stability of *ETS2* in UMUC3(shATG7) cells compared with UMUC3(Nonsense) cells. Upon inhibition of new mRNA transcription with Act D, the *ETS2* mRNA degradation rates in UMUC3(shATG7) cells were much more stable than in UMUC3(Nonsense) cells (Figure 7H), suggesting that ATG7 overexpression led to *ETS2* mRNA instability in BC cells. To determine whether ETS2 directly binds to the promoter region of miR-196 for inhibiting its transcription, a ChIP assay was performed by using anti-ETS2 antibody in UMUC3 cells. As shown in Figure 7I, ETS2 protein did form complexes with the motifs between −851 and −683 bp of the miR-196b promoter. Collectively, our results demonstrate that ATG7 overexpression leads to *ETS2* mRNA degradation, subsequently increases *miR-196b* transcription, and further decreases *FOXO1* mRNA stability by targeting its mRNA 3′ UTR, in turn inhibiting *p27* transcription and promoting G2/M phase transition and finally promoting the tumorigenic growth of human BC, as diagrammed in Figure 7J.

## Discussion

The canonical pathway controlling autophagosome formation requires ATG7, which acts as an E1 enzyme and is responsible for the two ubiquitin-like systems required for transport from the cytoplasm to the vacuole and further leading to autophagy.^27^, ^28^ In the current study, we define the status and role of ATG7 in the promotion of human BC tumorigenic growth and elucidate the ATG7 downstream effector(s) resulting in the regulation of BC tumorigenic growth. We show that ATG7 expression is remarkably upregulated in human BC tissues, which is consistent with the finding that ATG7 is overexpressed in BBN-treated human urothelial cells as well as BBN-induced mouse invasive BCs. We also define the positive promotion of ATG7 for anchorage-independent growth of human BC cells in vitro and tumorigenic growth in vivo. Our results further indicate that ATG7 overexpression reduces *ETS2* mRNA stability, consequently promoting *miR-196b* transcription, and *FOXO1* mRNA degradation, in turn inhibiting *p27* transcription and expression. Our data collectively provide a significant insight into the understanding of the nature of ATG7, implicated in the promotion of BC growth, which raises the potential of developing of ATG7-based specific therapeutic strategies for the treatment of human BC patients.

ATG7 has been reported to be associated with growth and invasion through multiple mechanisms in a few cancers.^4^, ^6^, ^29^ It has been reported that ATG7 can bind to the tumor suppressor TP53 and, in turn, regulates the transcription of p21 and, eventually, the activation of cell death pathways.^30^ ATG7 downregulation promotes the invasion of glioblastoma cells accompanied by an upregulation of the two epithelial-mesenchymal transition (EMT) regulators SNAI1 and SNAI2.^31^ On the other hand, positive regulatory effects of ATG7 on tumor growth and invasion have also been reported. For example, ATG7 deficiency strongly attenuates the growth of intestinal tumors, accompanied by metabolic defects, AMPK (5′ AMP-activated protein kinase) activation, and p53-mediated cell cycle arrest in a relevant preclinical animal tumor model.^32^ ATG7 deficiency inhibits the growth of Braf^V600E^-driven melanoma with increased oxidative stress and senescence.^33^ All of these studies strongly reveal that the specific biological effects of ATG7 on tumor growth and progression are tumor type-dependent. In agreement with ATG7 overexpression in BC cell lines in vitro and human BCs and mouse BCs in vivo, the current study indicates that knockdown of ATG7 leads to attenuation of BC cell anchorage-independent growth in vitro and tumorigenicity in vivo. These studies, together with our most recent finding that ATG7 is crucial for BC cell invasion (J.Z., unpublished data), provide the first evidence demonstrating the oncogenic role of ATG7 in BC. Therapeutically targeting the ATG7 protein is a highly attractive approach for BC treatment.

As a cyclin-dependent kinase inhibitor, p27 could negatively modulate cell cycle progression through inhibition of the G2/M phase.^34^, ^35^ Decreased p27 expression is reported to be associated with poor overall survival in patients with muscle-invasive BCs.^36^ Our current studies explored the mechanisms leading to p27 downregulation in BCs, which have not been fully understood before. The results indicate that ATG7 overexpression is at least a key upstream regulator for p27 downregulation in BCs. Moreover, we identify ATG7 overexpression results in reduction of FOXO1-dependent *p27* transcription, thereby inhibiting the tumorigenic growth of BC cells, which highlights the tumor suppressor function of p27 in human BC growth. However, an oncogenic function of p27 in driving tumor metastasis through the activation of EMT in human BC cells has previously been reported.^36^ Inhibition of p27 abolishes migration, invasion via decreasing the phosphorylation of ERK1/2, and expression of MMP-9 in interleukin-7 (IL-7)-stimulated human BC 5637 cells.^37^ These results reveal that the prognosis value of p27 depends on its downstream targets in a specific signaling axis and stage of BC cells. Our most recent studies demonstrate that ATG7 deficiency results in a dramatic inhibition of BC invasion abilities. So the novel finding of p27 in the regulation of ATG7-dependent tumorigenic growth in BCs raises the question of whether p27 mediates the effect of ATG7 overexpression on promotion of BC invasion. Further evaluation of this possibility will provide significant insights into the nature of p27 in BC.

FOXO1, a member of transcription factor FOXO family, is characterized by the presence of a winged helix DNA binding domain called a forkhead box.^38^ Microarray evidence has revealed that FOXO1 overexpression suppresses or increases the expression of essential G2/M transition genes, including *Cyclin D1*, *CDK2*, *CDC2*, *NEK2*, *p27*, and p21.^39^, ^40^, ^41^ The current study provides strong evidence that ATG7 overexpression downregulates FOXO1 expression in vitro and in vivo. Mechanistic studies reveal that FOXO1 functions as a transcription factor responsible for initiating *p27* transcription and expression and further induces G2/M arrest of human BC cells. FOXO1 serves as an ATG7 downstream effector for the promotion of BC tumorigenic growth. Such tumor suppressor properties of FOXO1 in human BCs are also in agreement with our recent findings that downregulation of FOXO1 expression promotes BC invasion abilities (G.J., unpublished data) and that the anti-cancer compound isorhapontigenin is able to elevate FOXO1 expression and inhibit invasion of human BC cells,^42^ suggesting that inhibition of FOXO1 might be of therapeutic benefit for human BC patients.

FOXO1 expression can be regulated at multiple levels, including mRNA transcription, mRNA stability, protein translation, and degradation. miRNAs, a class of small (21- to 23-nt) non-protein-coding RNAs, can regulate either mRNA stability or protein translation.^43^ miR-223 and miR-370 can directly target FOXO1 and regulate endogenous FOXO1 protein expression and are also responsible for cancer cell proliferation.^44^, ^45^ In the current study, ATG7 overexpression reduces *FOXO1* mRNA stability and protein expression. We also show that *FOXO1* 3′ UTR mRNA luciferase reporter activity is remarkably increased in ATG7 knockdown transfectants, and such an elevation could be completely impaired when the miR-196b binding site on the *FOXO1* 3′ UTR mRNA luciferase reporter is mutated. Moreover, we demonstrate that ectopic expression of miR-196b in UMUC3(shATG7) cells also inhibits FOXO1 and p27 expression, promotes *FOXO1* mRNA degradation, and further elevates the anchorage-independent growth of BC. We conclude that miR-196b exerts its oncogenic role in human BC cells by directly inhibiting FOXO1 expression.

In summary, our studies have revealed a new ETS2/miR-196b/FOXO1/p27 pathway that is responsible for the oncogenic role of ATG7 in BC tumorigenic growth in vitro and in vivo. ATG7 overexpression destabilizes *ETS2* mRNA, further elevates *miR-196b* transcription and expression, followed by impairing *FOXO1* mRNA stability by directly binding with its mRNA 3′ UTR, in turn inhibiting *p27* transcription and promoting G2/M phase transition and growth of human BC cells. Given our most recent discovery of the crucial role of ATG7 in promoting BC invasion, our new findings raise the potential for developing an ATG7-based specific therapeutic strategy for the treatment of human BC patients.

## Materials and Methods

### Plasmids, Antibodies, and Reagents

The constructs of short hairpin RNA specifically targeting ATG7 (shATG7), p27 (shp27), FOXO1 (shFOXO1), and NCL (shNCL) were purchased from OpenBiosystem. miR-196b, FLAG-ETS2, shETS2, and the plasmid of the *miR-196b* promoter were a kind gift from Dr. Wen-chang Lin (Institute of Biomedical Sciences, Academic Sinica).^26^ The human *FOXO1* promoter was cloned into the pGL3 basic luciferase reporter and was kindly provided by Dr. Jean-Baptiste Demoulin (De Duve Institute, Catholic University of Louvain).^46^ The human *FOXO1* mRNA 3′ UTR luciferase reporter was kindly provided by Dr. Bruce A. White (Departments of Cell Biology and Molecular, Microbial, and Structural Biology, University of Connecticut Health Center).^47^ The human *FOXO1* 3′ UTR mRNA mutant fragment (the binding site of miR-196b was mutated) was cloned into the pMIR luciferase reporter vector by *XhoI* and *NotI* restriction endonucleases. The *p27 KPNI* promoter luciferase (−1324 to +461) and *p27 SACII* promoter luciferase (+162 to +461) plasmids were kind gifts from Dr. Toshiyuki Sakai (Department of Molecular-Targeting Cancer Prevention, Graduate School of Medical Science, Kyoto Prefectural University of Medicine).^48^ Plasmids were prepared with the Plasmid Preparation/Extraction Maxi Kit from QIAGEN. The chemical Act D was purchased from Calbiochem. BBN (B0938) was purchased from TCI American.

The antibodies specific against ATG7, p27, Cyclin A2, Cyclin B1, FOXO1, c-Fos, c-Jun, p65, HuR, and GAPDH (glyceraldehyde 3-phosphate dehydrogenase) were purchased from Cell Signaling Technology. Antibodies against p53, E2F1, CDK2, Sp1, NCL, and ETS2 were bought from Santa Cruz Biotechnology. The antibody specific against AUF1 was purchased from Aviva Systems Biology. The antibody against β-actin was bought from Sigma.

### Cell Lines and Cell Culture

Human BC cell lines, UMUC3 and T24, were used and are described in our previous studies.^49^, ^50^, ^51^, ^52^ These cells were maintained in DMEM-F12 (1:1) (Invitrogen) supplemented with 5% heat-inactivated fetal bovine serum (FBS), 2 μM L-glutamine, and 25 μg/mL gentamycin. The human normal bladder urothelial cell line UROtsa was a gift from Dr. Scott Garrett (Department of Pathology, School of Medicine and Health Sciences, University of North Dakota)^53^ and was used in our previous publication.^54^ These cells were maintained at 37°C in a 5% CO_2_ incubator with RPMI 1640 medium supplemented with 10% FBS (26140079) and 2 mM L-glutamine (25030164). All cell lines were subjected to DNA tests and authenticated before/after utilization for research by Genetica DNA Laboratories using a PowerPlex 16 HS system.

### Construction of the *FOXO1* 3′ UTR mRNA Mutant Luciferase Reporter

A three-point mutation was introduced into the seed region of the *miR-196b/FOXO1* putative interacting sequence (Figure 6C) using primers MIRMUTFOR (5′-*GTT AAA CTT TTG TTT GTA CCG TGT GTT TTG TGC GGA ACT*-3′) and MIRMUTREV (5′-*AGT TCC GCA GAA AAC AGA CGG TAC AAA CAA AAG TTT AAC*-3′) according to the site-directed mutagenesis protocol (QuikChange Site-Directed Mutagenesis Kit, Stratagene) for producing the pMIR-*FOXO1* 3′ UTR mRNA mutant plasmid. All constructs were sequence-verified by GENEWIZ.

### Transfection and Luciferase Assay

Cell transfections were performed by using PolyJet DNA in vitro transfection reagent (SignaGen Laboratories) according to the manufacturer’s instructions. Surviving cells from the antibiotics selection were pooled as stable mass transfectants as described in our previous studies.^3^, ^51^, ^55^ For the determination of *p27* promoter-driven luciferase activity, *FOXO1* promoter-driven luciferase activity or *miR-196b* promoter-driven luciferase activity, UMUC3(Nonsense), UMUC3(shATG7#1), and UMUC3(shATG7#2) cells were each transiently co-transfected with pRL-TK together with the related promoter-driven luciferase reporter. 24 hr after transfection, luciferase activity was determined using a luciferase assay system kit (Promega). For the determination of *FOXO1* mRNA 3′ UTR activity, UMUC3(Nonsense), UMUC3(shATG7#1), and UMUC3(shATG7#2) cells were transiently transfected with pRL-TK together with *FOXO1* mRNA 3′ UTR luciferase reporter or *FOXO1* mRNA 3′ UTR mutant luciferase reporter. 24 hr after transfection, luciferase activity was determined using a luciferase assay system kit (Promega). The results were normalized by internal TK signal. All experiments were done in triplicate, and the results are expressed as mean ± SE.

### RT-PCR

Total RNA was extracted using TRIzol reagent (Invitrogen) as described in the manufacturer’s instructions. Total RNA (5.0 μg) was used for first-strand cDNA synthesis with oligo (dT) 20 primer by Super-Script First-Strand Synthesis system (Invitrogen). Specific primers (Invitrogen) were used for PCR amplification. The primers used in this study were as follows: human *p27* (forward, 5′-*ACC CGC CCG AGG AGG AAG ATG T*-3′; reverse, 5′-*GCG CGG GGG CCT GTA GTA GAA C*-3′), human *FOXO1* (forward, 5′-*AAC CTG GCA TTA CAG TTG GCC*-3′; reverse, 5′-*AAA TGC AGG AGG CAT GAC TAC GT*-3′), human *ETS2* (forward, 5′-*AGC GTC ACC TAC TGC TCT GTC A*-3′; reverse, 5′-*CCG TTG CAC ATC CAG CAA*-3′), and human *β-actin* (forward, 5′-*CTC CAT CCT GGC CTC GCT GT*-3′; reverse, 5′-*GCT GTC ACC TTC ACC GTT CC*-3′). The PCR products were analyzed by agarose gel. The densitometry analyses of the product bands were performed using ImageQuant 5.2 software (GE Healthcare).

### ChIP Assay

The ChIP assay was carried out as described in our previous publication by using reagents that were purchased from Millipore.^56^ Briefly, the indicated genomic DNA and proteins were cross-linked with 1% formaldehyde (Protocol, 245-684). The cross-linked cells were pelleted, resuspended in lysis buffer, and sonicated to generate 200- to 500-bp chromatin DNA fragments. After centrifugation, the supernatant fractions were diluted 10-fold and then incubated with anti-FOXO1 antibody (Cell Signaling Technology, C29H4), anti-ETS2 antibody (Santa Cruz Biotechnology, sc-351), or the control rabbit immunoglobulin G (IgG) (Santa Cruz Biotechnology, sc-2027) overnight at 4°C, respectively. The immune complex was captured by protein G-agarose (Santa Cruz Biotechnology, C1014) saturated with salmon sperm DNA (Upstate Biotechnology, 0606031838) and then eluted with elution buffer. DNA-protein crosslinking was reversed by heating overnight at 65°C. DNA was purified and subjected to PCR analysis. To specifically amplify the region containing the putative responsive elements on the human p27 promoter, PCR was performed with the following pair of primers: 5′-GCT CGC CAG TCC ATT TGA T-3′ and 5′-CTC GCA CGT TTG ACA TCT TTC-3′. To specifically amplify the region containing the putative responsive elements on the human miR-196b promoter, PCR was performed with the following pair of primers: 5′-TCA GTT TTA TGG CTT GCT AG-3′ and 5′-GTC ATC TGT GAC CCA GAC AC-3′. The PCR products were separated on 2% agarose gels and stained with ethidium bromide; the images were then scanned with a UV light.

### qRT-PCR for miRNA Assay

Cells were cultured as described under Western Blot Analysis. The cells were then used for total RNA extraction using the miRNeasy Mini Kit (QIAGEN). Total RNA (2.0 μg) was used for reverse transcription. The analysis of miRNA expression was carried out using the miScript PCR system (QIAGEN) and the 7900HT fast real-time PCR system (Applied Biosystems). The initial activation was performed at 95°C for 15 min, followed by 40 cycles of denaturation at 95°C for 15 s, annealing at 55°C for 30 s, and extension at 70°C for 30 s. Data were analyzed as described in a previous publication.^57^

### Western Blot Analysis

UMUC3 cells and T24 cells and their transfectants were seeded in 6-well plates and cultured in normal medium until 70%–80% confluence. Following culture of cells in 0.1% FBS medium for 12 hr, the medium was replaced with 10% FBS DMEM or 5% FBS DMEM-F12 (1:1) for another 12 hr. UROtsa cells were treated with BBN at different doses for 24 hr or 1 month. Whole-cell extracts were prepared with cell lysis buffer (10 mM Tris-HCl (pH 7.4), 1% SDS, and 1 mM Na_3_VO_4_) as described in our previous studies.^58^, ^59^ Cell extracts were then subjected to western blot analysis as described previously.^60^, ^61^ Images were acquired by scanning with the phosphorimager (Typhoon FLA 7000, GE Healthcare).

### Cell Proliferation Analysis

Cell viability was determined by utilizing the CellTiter-Glo Luminescent Cell Viability Assay Kit (Promega) according to the manufacturer’s instructions. Briefly, cells were plated in 96-well plates at a density of 5,000 cells/well and allowed to adhere overnight. The cell culture medium was then replaced with 0.1% FBS DMEM or 0.1% FBS DMEM-F12 (1:1) and cultured for 12 hr. and the medium was then replaced with normal medium and cultured for another 1, 2, 3, or 4 days, and then 50 μL CellTiter-Glo assay reagent was added to each well. The contents were mixed on an orbital shaker for 2 min to induce cell lysis and then incubated at room temperature for 10 min to stabilize the luminescent signal. Results were read on a microplate luminometer (LB 96V, Berthold). Cell viability (percent) was defined as the relative absorbance of treated samples versus that of the untreated control. All experiments were performed in 96-well plates for each experiment and repeated at least three times.

### Cell Cycle Analysis

The indicated cells (2 × 10^5^) were cultured in each well of 6-well plates to 70%–80% confluence with normal culture medium. Following serum starvation for 12 hr, the medium was replaced with 10% FBS DMEM or 5% FBS DMEM-F12 (1:1) for another 12 hr. Then the cells were harvested and fixed with 3 mL of ice-cold 80% ethanol overnight. The fixed cells were then centrifuged (3,000 rpm, 3 min), suspended in lysis buffer (100 mM sodium citrate and 0.1% Triton X-100), and incubated for 15 min at room temperature. Then the cells were incubated with RNase A (10 μg/mL) (Sigma) for 10 min at room temperature, and DNA was stained with propidium iodide (50 μg/mL) for at least 1 hr at 4°C. The DNA content was determined by flow cytometry using Epics XL FACS (Beckman Coulter) and EXPO 32 software.

### Anchorage-Independent Growth Assay

Anchorage-independent growth in soft agar (soft agar assay) was performed as described in our earlier studies.^61^, ^62^ Briefly, 1 ×× 10^4^ cells in 10% FBS basal medium Eagle (BME) containing 0.33% soft agar were seeded over the basal layer containing 0.5% agar in 10% FBS BME in each well of 6-well plates. The plates were incubated in a 5% CO_2_ incubator at 37°C for 3 weeks. Colonies were captured under a microscope, and only colonies with over 32 cells were counted. The results are presented as mean ± SD obtained from three independent experiments.

### Human BC Tissue Specimens

12 pairs of primary invasive BC samples and their paired adjacent normal bladder tissues were obtained from patients who underwent radical cystectomy at the Department of Urology of the Union Hospital of Tongji Medical College between 2012 and 2013. All specimens were obtained with appropriate informed consent from the patients and a supportive grant obtained from the Medical Ethics Committee in China, and all work was carried out in accordance with The Code of Ethics of the World Medical Association (Declaration of Helsinki) for experiments involving humans. All specimens were immediately snap-frozen in liquid nitrogen after surgical removal. Histological and pathological diagnoses were confirmed, and the specimens were classified by a certified clinical pathologist according to the 2004 World Health Organization Consensus Classification and Staging System for bladder neoplasms.^42^

### In Vivo BBN Treatment of Mice and Tumor Xenografts

All animal procedures were approved by the University Committee on Animal Resources of New York University in accordance with NIH guidelines. C57BL/6J mice at an age of 3∼4 weeks were randomly divided into two groups, including a negative control and BBN group. In the BBN group, each mouse was supplied ad libitum with tap water containing 0.05% BBN in opaque bottles for 23 weeks. The drinking water was freshly prepared twice a week, and consumption was recorded to estimate BBN intake. Negative control mice received regular tap water. The mice from the BBN group and control group were sacrificed after the last BBN exposure. The bladder tissues were removed for pathological analysis and evaluation of the expression of ATG7 with IHC staining. The tumor xenograft studies were performed in the Animal Institute of Wenzhou Medical University according to the protocols approved by the Medical Experimental Animal Care Commission of Wenzhou Medical University. Fifteen female athymic nude mice (3–4 weeks old) were purchased from Shanghai Silaike Experimental Animal Company (license no. SCXK, Shanghai 20100002), and the mice, at an age of 5–6 weeks, were randomly divided into different groups as indicated and then subcutaneously injected with various UMUC3 transfectants (2 × 10^6^ suspended in 100 μL PBS) in the axillary region. The nude mice were maintained under sterile conditions according to the protocol of the American Association for the Accreditation of Laboratory Animal Care. These mice were evaluated twice a week for the appearance and size of tumors, and tumors were measured with calipers to estimate the volume. Tumor sizes were evaluated using the following formula: volume (mm^3^) = (width2 [mm^2^] × length [mm])/2. Four weeks after cell injection, the mice were sacrificed, and the tumors were surgically removed, photographed, weighed, and used for further pathological and histopathological evaluation. None of the mice died or were sacrificed before the end of the in vivo experiment.

### IHC Paraffin Embedding of Human Bladder Specimens

Tumor tissues obtained from the sacrificed mice were formalin-fixed and paraffin-embedded. For the IHC assay, we used antibodies specific against FOXO1 (Cell Signaling Technology) or p27 (Cell Signaling Technology). The resultant immunostaining images were captured using the AxioVision Rel.4.6 computerized image analysis system (Carl Zeiss). Protein expression levels were analyzed by calculating the integrated optical density (IOD) per stained area using Image-Pro Plus version 6.0 (Media Cybernetics).

### Statistical Analysis

Statistical analysis was performed using Prism 5.0 software (GraphPad). All data are presented as the means of triplicate assays ± SD. Student’s t test was employed to determine the significance of differences between various groups. The differences were considered significant at p < 0.05.

## Author Contributions

C.H. and J.Z. conceived and designed the study. Y.L., H.H., J.L., Y.W., H.J., X.H., and J.Z. detected the cells’ biological function, conducted the RT-PCR assays, carried out the western blot and luciferase reporter assays, and performed the statistical analysis. Z.T. and J.G. carried out the animal studies and the IHC staining assays. G.J. provided the human bladder cancer tissues specimens. C.H., J.Z., and C.L. drafted the manuscript. All authors read and approved the final manuscript.

## Conflicts of Interest

The authors declare no conflict of interest.
